# Development and Validation of a Photographic Method to Use for Dietary Assessment in School Settings

**DOI:** 10.1371/journal.pone.0163970

**Published:** 2016-10-06

**Authors:** Anna S. Olafsdottir, Agneta Hörnell, Marlene Hedelin, Maria Waling, Ingibjörg Gunnarsdottir, Cecilia Olsson

**Affiliations:** 1 School of Education, University of Iceland, Reykjavik, Iceland; 2 Department of Food and Nutrition, Umeå University, Umeå, Sweden; 3 Unit for Nutrition Research, Landspitali -The National University Hospital of Iceland and Faculty of Food Science and Nutrition, School of Health Sciences, University of Iceland, Reykjavík, Iceland; Universitat de Lleida-IRBLLEIDA, SPAIN

## Abstract

**Objective:**

To develop and validate a photographic method aimed at making assessment of dietary intake in school canteens non-obstrusive, practical and feasible.

**Methods:**

The study was conducted in two elementary schools representing two different school canteen systems; main dish being served by canteen staff (Iceland), and complete self-serving (Sweden). Food items in serving and leftovers were weighed and photographed. Trained researchers estimated weights of food items by viewing the photographs and comparing them with pictures of half and full reference portions with known weights. Plates of servings and leftovers from 48 children during five school days (n = 448 plates) and a total of 5967 food items were estimated. The researchers’ estimates were then compared with the true weight of the foods and the energy content calculated.

**Results:**

Weighed and estimated amounts correlated across meals both in grams and as total energy (0.853–0.977, p<0.001). The agreement between estimated energy content in school meals was close to the true measurement from weighed records; on average 4–19 kcal below true values. Organisation of meal service impacted the efficacy of the method as seen in the difference between countries; with Iceland (served by canteen staff) having higher rate of acceptable estimates than Sweden (self-serving), being 95% vs 73% for total amount (g) in serving. Iceland more often had serving size between or above the half and full reference plates compared with Sweden.

**Conclusions:**

The photographic method provides acceptable estimates of food and energy intake in school canteens. However, greater accuracy can be expected when foods are served by canteen staff compared with self-serving.

## Introduction

Diet can be assessed in many ways, and the selection of methodology depends on the nature of information needed. Common methods used to investigate dietary intake are food records, dietary recalls, food frequency questionnaires (FFQs), and diet history interviews [1]. All these methods have their strengths, but also limitations, inborn errors and difficulties that increase the risk for misreporting. Since these dietary survey methods depend on self-reporting, one of the major sources of error is difficulty in assessing portion sizes [2]. Several studies demonstrate a tendency towards underreporting [2, 3] and foods may also be forgotten with or without intention.

A recent study found that children classified as under-reporters, reported less frequent consumption as well as smaller quantities of many food types compared to plausible reporters [4]. Notably, these foods comprised both core (e.g. bread, milk, cheese, fruit and vegetables) and noncore foods (e.g. mostly energy-dense and nutrient poor foods such as cakes, sweets, crisps and sugar-sweetened beverages). It was not clear whether these foods were purposely underreported, forgotten or under-eaten among younger children (2–8 years old; parental report), but among older children (9–16 years old; self-reported intake) a selective bias towards the underreporting of noncore foods was observed, while fewer core foods were underreported.

Apart from the general limits of most dietary assessment methods, assessing children’s diet encounters particular challenges. Young children have not developed the cognitive systems required to assess portion sizes appropriately [5, 6]. Also literacy skills needed for proper self-reporting methods might be limited and motivation may be lacking [7]. Studies indicate that different age groups require different methods to best assess dietary intake. In general, children over 12 years of age have developed the required cognitive skills to give reliable reports of their own intake [2]. Studies validated by using the doubly labelled water (DLW) method have however found that for children 4–11 years of age, repeated 24-hour recalls by parents, including at least three days and both weekdays and weekends, seem to best reflect actual energy intake [8]. When assessing dietary intake in schools, parents are usually out of reach and it cannot be expected that assistance and supervision from adults are available.

Direct observations where any self-reporting is excluded have shown good correlation with weighing methods and have only slight over- and underestimations [9]. Such observations are non-obtrusive and well suited to cafeteria settings. A disadvantage with observations is however that they are time consuming for the researchers. Consequently there is a need for dietary assessment methods that neither are dependent on participants’ memory nor are time consuming, cumbersome or obtrusive. As such, photographic methods have gained attention and popularity, and a diverse range of image-assisted methods have been developed [10].

Studies using photographic methods as a tool for estimating energy intake have indicated valid and reliable results for both adults and children [11–14]. In addition, Martin et al. also display good inter-agreement between trained observers [1]. Further photographic methods can overcome some of the problems with self-reporting methods, since they are not dependent on respondents cognition and moreover are of little burden, non-obtrusive and time effective [13]. Research on the validity of photographic methods among children and adolescents, as well as the feasibility of such methods in large scale studies, is being called for [10, 15], especially practical methods that can assess school lunch intake [16].

The aim of this study was to validate a photographic method for use in school/canteen settings, where children’s intake of food, certain food items and energy intake can be estimated from photographs by comparison with photographs of weighed reference portions.

## Materials and Methods

The validation study was conducted in two elementary schools; one in Iceland and one in Sweden as a part of preparations for a Nordic multi-centre study on school meals conducted 2013/14 (*Prospects for promoting health and performance by school meals in Nordic countries; ProMeal*)[17]. The ProMeal study protocols including consent procedure were approved by ethical boards in the participating countries; In Iceland by The National Bioethics Committee (56363) and The Icelandic Data Protection Authority (VSN-13-088) and in Sweden by The Regional Research Ethics Review Board, the Faculty of Medicine, Umeå University (2013-212-31Ö). The schools for the validation study were chosen from those participating in ProMeal after agreement with principals and teachers. Written consent was gathered from the caretakers/parents of the participating children and an oral consent from the children themselves. In total 19 ten-year-old children from Iceland and 29 children from Sweden participated in the validation study.

No personal data was collected during the validation study since results are only based on the amount of foods and leftovers put on each child’s plate. The children got numbered trays in order for us to be able to match taken servings with leftovers but the children were not identified in any way.

The countries represented two different school canteen systems; main dish being served by canteen staff (Iceland), and complete self-serving (Sweden). In Iceland, the children waited in line to receive the main dish. The canteen-staff asked the children how large portions they wanted and if they wanted to have all items of the main dish. The children then served themselves as much vegetables as they liked and one piece of fruit from a side table. In Sweden, the children also stood in line, but served themselves each food component from large containers. There was also a separate line for a salad bar including a variety of vegetables each day, and fruit on some days.

### Data collection

Data were collected during five school days; in Iceland the days were chosen in order to get diverse foods including the most common school meals, but in Sweden the days were consecutive. Photographs were taken during the regular lunchtime in the respective school canteens (Canon EOS650D in Iceland and Sony NEX-5R in Sweden).

For each day, half and full reference portions of the meal were created by canteen staff in each country. These were weighed and photographed according to protocol. The plates were placed on trays and photographed with two cameras mounted on tripods over a table in such a way that photographs could be made above the tray (90° angle) and at a 45° angle for perception of depth. Both photos were taken simultaneously with a remote controlled shutter release. Cards with identification numbers were put on the trays in order to connect them with weighing protocols. The purpose of the reference portions was to serve as a guide when estimating the amount of food on the children’s plates. A reference booklet was produced, one for each country, with pictures of all the reference portions and with the weights of each food item written next to them (available as supplementary file). The reference booklet was available both in paper format and on computer screen.

For the actual meals, each food item was weighed separately by trained research assistants as it was placed on the plates. Where possible, all plates were turned the same way, i.e. with the same food placement on each photograph, since the position of the food on the plate may influence the sense of the portion size. For leftovers, proceedings were reversed, i.e. the photographs were taken before the food was weighed and then finally discarded.

### Estimation of portion sizes

**Fig 1:**
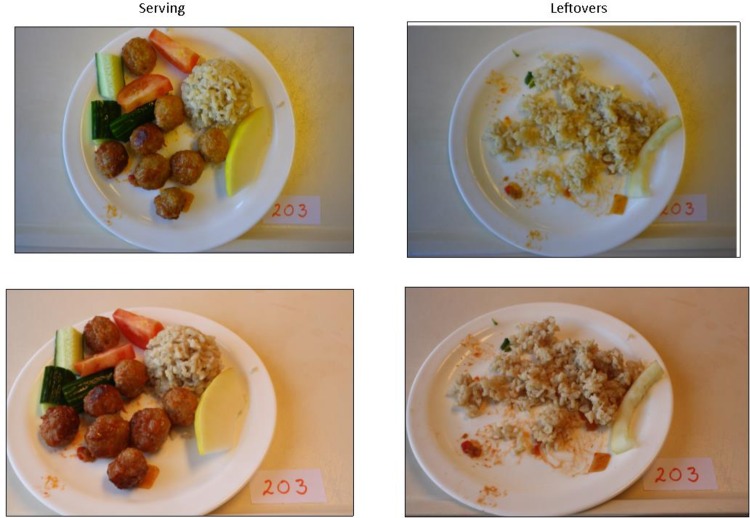
Example of served and leftover plates of the same meal. Serving left, leftovers right.

In each country, four trained researchers, different from those weighing the food and hereafter called ‘estimators’, got a copy of all plate photos to view on computer screen; including both angles, but without their weights (Fig 1). The weights of each food item in the photographs were estimated in grams, by comparison with the weights given in the reference booklet, and written on separate sheets for each estimator. Estimations were done in the same manner for leftovers. Estimation of the weight of food items like cinnamon-sugar to porridge or sauce in casseroles were calculated as a proportion of the meal they came with.

### Data handling

Based on the total number of children participating (n = 48), the expected number of plates were 480 (5 days of datacollection with both serving and leftovers), but not all children were attending on all school days. Also sometimes they forgot to bring back their plates for weighing and photographing of leftovers.

Data from the weighings and estimations were combined into a protocol together with information about the weighed amount of the half and full reference portions, for comparison of estimated weights from each estimator with the true weight. Only food taken in the serving were included in the analysis.With many foods being present on each plate, sometimes estimators forgot to estimate some of the items on a given plate leaving them as missing in analysis. As a result, total number of servings differs between type of food as well as serving and leftovers.

To test the feasibility of the method for different types of foods, food items were grouped depending on nutrient content and/or shape. Seven groups were formed: 1) *carbohydrate-rich foods* (e.g. rice, potatoes, pasta, bread); 2) *protein-rich foods* (e.g. fish, meat and poultry of any kind); 3) *mixed dishes* (i.e. rich in both carbohydrate and protein, e.g. pancake, lasagna, rice pudding; 4) *amorphous foods* (i.e. food items that lacked a definite form or clear shape, e.g. casseroles, rice, shredded vegetables); 5) *large pieces* (i.e. food items that could be counted, e.g. fish fingers, meatballs, potatoes, fruit and vegetables cut in big pieces); 6) *small pieces* (i.e. difficult to count but still visible as pieces, e.g. diced pepper, beans, sweet corn), and 7) *fruit and vegetables*. Most of the food items were included in more than one group.

**Fig 2:**
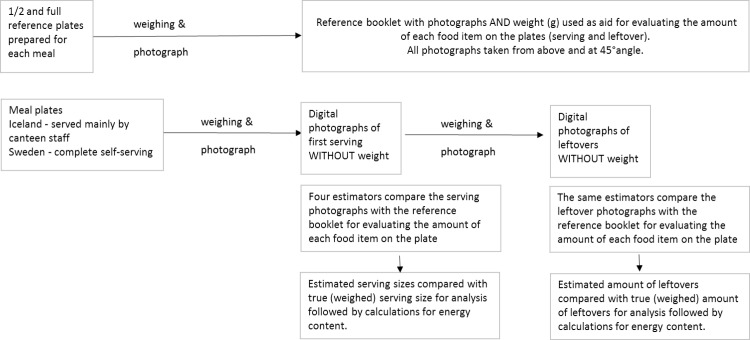
Study design–procedure in each country.

The nutritional content of both reference portions and estimates was calculated using the nutrient calculating program ICEFOOD (v.2.0) in Iceland and Dietist Net Pro (version 15.02.14) in Sweden. Calculations were based on recipes and reference portions from each canteen. Overview of study design and data handling is shown in Fig 2.

To assess the applicability of the method, the agreements of the photo method with the weighted amounts (g) and the calculated energy content (kcal), were divided into three groups depending on how close the estimate was to the weighed amount. Different approaches and limits of agreement have been used in previous validation studies [13, 18, 19]. In the present study, an estimate between ± 25% from the actual weight was considered an acceptable estimate, while estimates below -25% and above +25% were considered as underestimate and overestimate, respectively. Categorization for kcal was performed in the same manner.

To assess whether portion size affected the usability of the method, each weighed food item (from serving and leftovers separately) was categorized into being lower than the half reference plate, between the half and full reference plates, or above the full reference plate. For these analyses all non-present (zero) items were excluded. The proportion of under-, acceptable- and overestimations were then calculated for each portion size.

### Statistical analysis

To examine if our method ranks correctly when compared with the weighed amounts, Spearman correlation coefficients between the photo method and the weighed method were calculated for all meals (grams and kcal), with serving and leftovers combined as well as separately. To examine the agreement between the two methods, modified Bland–Altman plots [20, 21] were used. The weighed method was considered a golden standard since weighing is the most accurate method for measuring food intake [9]. Limits of the agreement between the methods were calculated as the mean difference between the two methods ± 2 standard deviations (2SD), shown as dotted lines (y-axis). Plots were made separately for each country and presented as servings and leftovers, and the amounts of food were described either in grams or energy (kcal). The total number of observations for all five days and four observers in each country were included in each plot.

Inter-rater variability in each country was determined for all of the foods by calculation of intra-class correlation coefficients (ICC).

Chi-squared analysis was used for country comparison of estimation accuracy (in categories of acceptable-, over- and under-estimates): 1) overall estimation accuracy for meals; 2) agreement of servings and leftovers with weight portions by type of foods, and 3) as a comparison of the weighed serving and leftovers in relation to amount on reference plates.

Data were prepared and analyzed using Excel (Redmond, Washington: Microsoft, 2010) and SPSS, version 22.0 (IBM Corp., Armonk, NY, USA). P-values <0.05 were considered significant.

## Results

In the present study we used photographs to estimate the amounts of 57 food items on 448 plates with servings or leftovers from ten different school meals in two schools. Because each plate had multiple items on it and each plate was evaluated by four estimators the total number of estimations was 5967.

Correlations for total gram of food and total energy on all plates for each of the five days were all high (r = 0.853–0.977, p<0.001 and r = 0.874–9.966, p<0.001, for Iceland and Sweden respectively).

**Fig 3:**
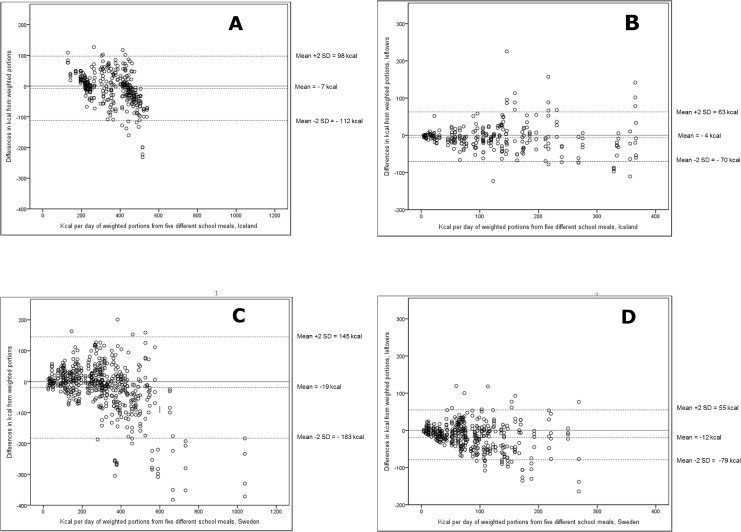
Difference between the observed and the weighed amount by serving method (country)—Modified Bland-Altman. a) Total energy (kcal) content in served meal, Iceland (served by canteen staff); b) Total energy (kcal) content in leftovers, Iceland; c) Total energy (kcal) content in served meal, Sweden (selfserving); d) Total energy (kcal) content in leftovers, Sweden.

In Fig 3A–3D mean bias and limits of agreement as mean +/- 2SD and their respective values are shown with three dotted lines on each figure, for servings and leftover for both countries separately. The modified Bland-Altman plots indicated a slight bias towards underestimation with the mean of the photo method ranging from 4 to 19 kcal below the true mean from the weighed portions (p<0.05 for all findings) for leftovers in Iceland having the smallest bias (-4 kcal; Fig 3B) and servings in Sweden the largest bias (-19 kcal; Fig 3C), respectively. Larger deviations from the mean were observed with increasing amounts (kcal) on the plates. Limits of agreement, reflecting the precision of the method, were approximately the same for leftovers in both countries, with a range of 134 kcal for Iceland and 133 kcal for Sweden respectively (Fig 3B and 3D), while being broader for servings (200 kcal for Iceland and 328 kcal for Sweden, respectively; Fig 3A and 3C). Considering the bias as % of kcal in each meal there was no significant mean bias for serving in neither country while the differences in leftovers deviated more from the true weighed values in both countries (-12% [95% CI -16%, -7%] for Iceland and -18% [95% CI -22%, -13%] for Sweden respecticely).

Inter-rater reliability between estimators in each country showed high ICC (>0.90) for all foods except tomato salsa (ICC = 0.80; 95%CI 0.70–0.87) and bean salad (ICC = 0.74; 95%CI 0.61–0.83) in Sweden.

Iceland had a significantly higher rate of acceptable estimates, with an average of 73% of all food items being within the acceptable range when looking at serving and 72% for leftovers, compared with Sweden with an average of 66% for servings and 65% for leftovers (p<0.001; data not shown). Iceland showed a 95% agreement for total amount in serving, but estimates of energy per meal were slightly lower and lowest for leftovers, with 69% within the acceptable range (p<0.001; Table 1). Proportions within acceptable estimates were significantly lower for Sweden, ranging from being highest at 73% for energy per meal in serving and lowest at 53% acceptable estimates for energy from leftovers.

**Table 1:** Agreement^1^ of amount (g) and energy content (kcal) estimated by photomethod with weighed method—by country.

	Served/taken food				Leftovers			
	Total plates estimated^2^	Under-estimated	Acceptable estimation	Over-estimated	Total plates estimated^2^	Under-estimated	Acceptable estimation	Over-estimated
Type of food	n	%	%	%	n	%	%	%
Iceland								
amount per meal (g)	344	2	95	3	316	17	71	12
energy per meal (kcal)	344	4	92	5	316	20	69	11
Sweden								
amount per meal (g)	532	15	73	12	510	35	53	12
energy per meal (kcal)	532	14	73	13	510	34	53	13

^1^ Underestimated = below -25% of true value; acceptable estimation = within ±25% of true value; overestimation = above +25% of true value

^2^For each country there were five different meals; number of servings/leftovers are based on the number of plates observed.

**Fig 4:**
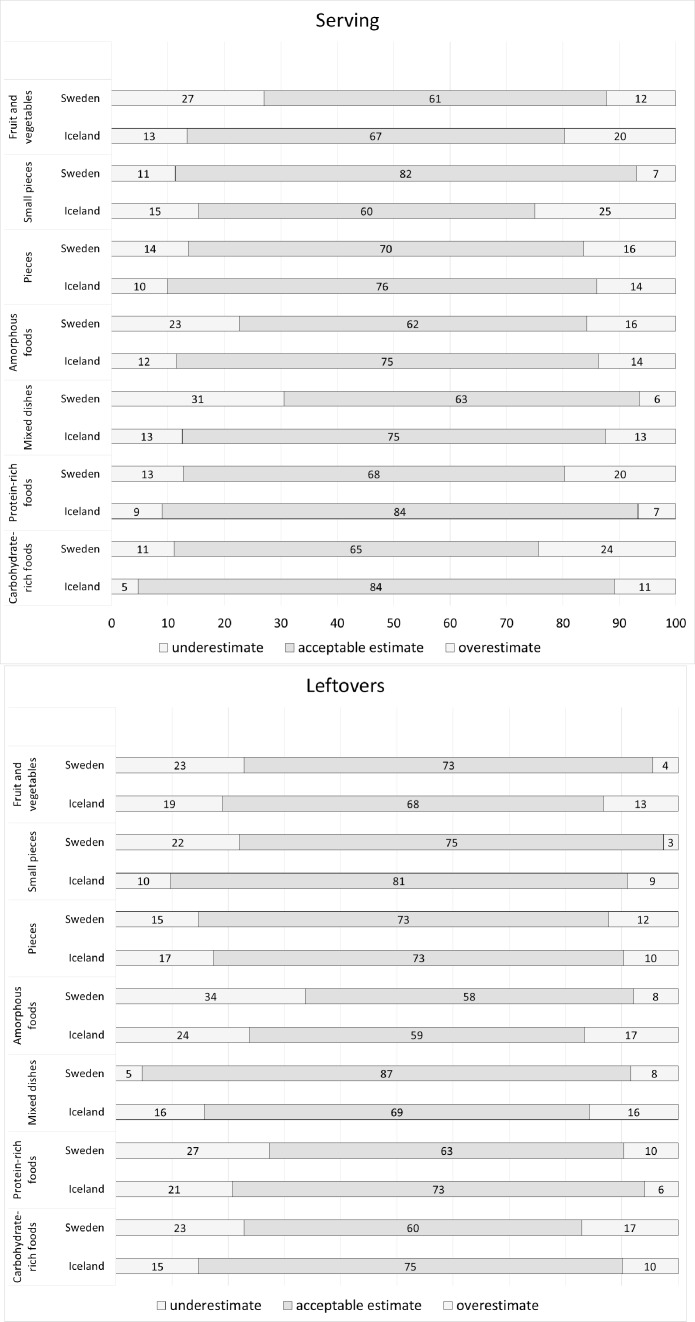
Agreement of photo method with weighted portions by country and type of foods (%). Dark part of column represents the proportion of acceptable estimates, with lighter part of column showing under- and overestimate respectively. a) Serving, b) Leftovers. Estimation of all food items/groups was significantly different (p<0.05) between the countries except for leftovers of pieces (p = 0.175).

The proportion of acceptable estimates for weighed portions of different food items were highest in Iceland in all but the estimation of *small pieces* (Fig 4A); p<0.05 for all comparisons. In Iceland, best estimates were reached for *carbohydrate-rich foods* and *protein-rich foods* (both 84% acceptable estimates), while Sweden showed the highest acceptable estimate for *small pieces* (82%), with the group *pieces* second best at 70% acceptable estimates. In six of the seven food groups, Iceland had the smallest proportion of under-estimation, varying between 5–13% underestimation, while Sweden underestimated 11–31% of the servings. The overestimated proportion was more similar and varied between 7–25% for Iceland and 7–24% for Sweden.

Estimation was significantly different between countries also for leftovers; except of *pieces* (p = 0.175; Fig 4B). The differences between the countries were smaller for leftovers, although Iceland had less underestimation also here in five of the seven food groups. The underestimation in Iceland varied between 10–24%, and in Sweden between 5–34%. *Amorphous foods* were the most underestimated in both countries. Best estimates in the two countries were made for *small pieces* in Iceland (81% acceptable), and *mixed dishes* in Sweden (87% acceptable). The overestimated proportion was more similar also for leftovers and varied between 6–16% for Iceland and 4–17% for Sweden.

Comparison between the weighed portions and corresponding half and full reference plates showed that Iceland had larger sizes of both servings and leftovers compared with Sweden. In Iceland, 18% of the servings were smaller than half reference portion, 51% were between the two visual aids, while 31% were larger than the full reference portion. In contrast, corresponding numbers in Sweden were 71%,19% and 10% (p<0.001 for country comparison). Comparable calculations for leftovers (not including plates if food items were fully consumed) showed 63% of the leftovers in Iceland to be less than half reference plate and 31% between half and full, while in Sweden 80% were smaller than the half reference plate and 17% were between half to full reference plates (p<0.001 for country comparison).

## Discussion

The photo method developed for the Promeal project was aimed at making assessment of dietary intake during school hours in school canteens practical and feasible. Bland-Altman plots showed that the agreement between estimated weights and energy content in school meals came close to the true measurement from weighed records; on average only 3–14 g and 4–19 kcal, respectively, below the true values. These findings suggest a relatively accurate estimate being achieved by the photo method. The limits of agreement refering to the precision of the method however indicate that meals being served by canteen staff may give more precise findings than self-servings, mainly due to more variability in portion sizes when children serve themselves. It is also notable that the bias of the method grew with bigger portions in both countries. Also strong correlations between weighed and estimated amounts were found across meals both in grams and when calculated as total energy, in line with what others have found [9]. Spearman coefficients use rank order and are less sensitive to extreme values while they implicate the value of the method for correctly ranking individuals according to their level of intake [22]. A recent systematic review also suggests that when used as primary record of dietary intake, photographs can provide valid estimates of energy intake if images taken before consumption are of satisfactory quality [10].

### The impact of meal service organisation

The organisation of the meal service had a strong impact on the efficacy of the method and thus on the differences seen between the countries. When the food was partly served by canteen staff (Iceland), acceptable estimates (±25% of the true value) were reached for 95% of all servings if viewed as total grams in meal and when calculated as energy it was true for 92% of the servings. In contrast, acceptable estimates were reached for 73% of all servings in the self-serving setting in Sweden. Leftovers were more difficult to assess than the servings in both countries. Overall, inter-rater reliability between estimators was high (>0.90) in both countries which supports the reliability of the method. This is in line with previous findings from a study of Williamson et al [9], although their study was conducted in a laboratory setting. Others have also shown ICC to be above 0.90 when trained registered dietitians have used photo technique for assessment of portion sizes [11] stressing the importance of using trained personell for this kind of studies.

The explanation for the effect of the organisation of the meal service, is that it affected the amount served as well as how the food was placed on the plate, which both had a bearing for the comparison with the reference pictures. In Iceland where the children partly got served by canteen staff in the schools, the amount of each food item was often similar between plates and there were few items missing from the plate, which may affect the perception of volume and thus the estimated amount of each food item. Also when served by canteen staff the food was more likely to be arranged in the same way on each plate, making each picture more easily compareable with the references. In Sweden, the amounts on the plates varied more and in addition the children had an ample choice of diverse vegetables everyday since they had a salad bar and not all of the available items were put on the reference plates for every day. The evaluation of amounts of these vegetables were therefore often based on comparison with pictures where the vegetables were presented in combination with another main meal and did not necessarily replicate the reference pictures as closely.

When children have the option of self-serving it may result in food not only being missing from the plate but also being less organised on the plate making the photo assessment more difficult since food may actually be hidden from the camera view. Leftover plates in both countries were also quite different from the reference pictures which might explain why acceptable estimates were less often achieved for them compared with serving plates. How this compares with the laboratory setting of Williamson et al.[9] is unclear. In their study, ten different portion sizes (0%-235% of reference) of foods from six different menus (total 60 test meals) were compared with reference portions.

As the different results from the two countries implicate, the method is of stronger value where the food choice in each meal is limited and at least some of the foods are served by the canteen staff.

### The impact of food type and portion size on estimations

The sizes of food items in the reference booklet of reference plates may cause some of the bias in the estimation of the amount on the children’s plates. This is especially true for the food group *pieces*, unless the pieces had a standardised size, since the estimators may have been counting rather than scrutinising the exact size of each piece.

It is also important to have procedures clear at the start of estimating the plates. For instance how to treat foods with peel, i.e. if the weight of the edible amount or weight with peel should be put into the protocol. In line with earlier findings [16], fruit can be difficult to assess since it may be taken out of the canteen without being eaten and therefore may be missing from the leftover photographs. We also had the experience of children consuming the (supposedly) inedible parts of the fruit.

Amorphous foods without a special shape and some mixed dishes tended to be the most difficult to estimate, and more so on the leftover plates. Other items being difficult to assess were small and light items such as leafy vegetables. Our results are much in line with other studies on the topic. Swanson found ground beef and tortilla chips (i.e. amorphous and light) to be most difficult to assess and also noted that because children often mixed these items visual estimation got more difficult and less consistent [16]. After being mixed up on the plate during eating some items in our study even looked as if there was more of them after eating than before the meal started. Due to the mixing and placement of items it was sometimes even difficult to detect what was on the leftover plate (e.g. due to smiliraties in colour and/or shape) resulting in misinterpretation among estimators. This was especially true if little was eaten, while empty plates were easy to estimate. In many cases, especially for fruit and vegetables in Sweden, there was a high proportion of non-takers. To avoid/minimize this type of bias, it is important for the precision of the method to simultaneously consider the serving and leftoverplates from each individual in relation to the reference portions.

### Additional aids when assessing dietary intake with photographs

Photographs are not equipped to stand alone in dietary assessments since they will not enable detection of cooking methods and hidden ingredients, and therefore it has been suggested that they must be supported by additional dietary information [10]. Our method covers these aspects since it relies on the recipes and reference portions from each canteen. In such a setting it is not necessary to put any burden on the participants with additional protocols of the foods being consumed. However, some foods may be almost impossible to accurately estimate by the use of photographs. Condiments such as ketchup, salad dressing and spread was excluded in a similar study [16], and others have found estimated weight of condiments to have the worst correlations with actual weights when using photographs for estimating diverse types of foods [9, 23]. Thus we based our estimates and energy calculations of food items like cinnamon-sugar to porridge or sauce in casseroles as a proportion of the meal they came with.

Other photo methods have been developed for different uses. For example by using digital images sent to registered dietians by mobile device for analysis [24]. A study presenting a similar photo method to the present study, but with the addition of a recall, was recently published showing promising results when compared with doubly labelled water [15]. The photo method presented here provides reasonable estimates of amount of foods and energy intake during school meals and furthermore the use of photographs allows for detailed information on what children put on their plates and what is left at the end of the meal. It is important to develop methods that are valid but not too burdensome for participants and investigators [15]. Collection of photographs is rapid and does not disrupt the busy cafeteria/school setting too much, where time constraints often are an issue [16]. It also gives the estimators ample time to consider the amounts.

### Value of not relying on self-report

Another point may be the importance and value of using photographs for detecting which foods are being eaten and as such the use of photographs should be promoted. As Swanson has pointed out, digital photography offers researchers and school food service personel a highly accurate and cost-effective tool to measure actual consumption, and data can be evaluated by simple counts of food groups or by more detailed analysis [16]. Several studies have demonstrated the strength of photographic methods to reveal underreporting, and to avoid misreporting and random errors that are common in traditional methods. Additionally, photographs avoid the pitfalls of self-reports since images increase objectivity [10]. Also, it is likely that trained staff are better at estimating amounts from photos than lay persons would be if asked to estimate how much they have eaten.

A strength of our study is the size of the sample (number of estimations) and the weighing of all plates being estimated in order to have a comparison with the true value. Few photographic methods have been formally validated using criterion measures and adequately big samples [10]. Furthermore the use of the reference pictures gives the method an added value since they are low in cost and easy to create. Such pictures may be especially advantageous if there is a predefined setting where available food items are not too many on any given day. It may also be assumed that the reference pictures are of more value if they show amounts similar to what is being served/taken. We found half of the plates in Iceland to be between half and full reference plate while less than 20% of servings in Sweden showed amounts between the two plates. This may be the reason why higher levels of agreement between estimated and true weight were achieved for the method in Iceland compared with Sweden. In a recent study using a similar photo method, research staff practiced estimating food items and portion size from sample pre- and post-meal photographs [15], although no reference photographs were used. In our study the estimators were all knowledgeable about foods and portion sizes. We found no systematic estimator bias, but, although not studied in a systematic way, the estimators stated that their confidence increased with growing numbers of photographs they went through, emphasizing the importance of pre-training when using the method. With experienced estimators the process was fast, and generally photographic methods are less time consuming than other means of collecting nutrition data [24]. Our method may therefore be useful even without the use of reference plates if the estimators are experienced. Other aids; for example household measures, predefined reference sizes [14], and more general food portion photograph books with weight information[25], might also be useful, either in combination with the use of reference plates or instead of them, especially if other foods are being eaten than served in the canteen.

### Statistical methods used for validation studies

Correlation coefficients are commonly used in validation studies on dietary intake since they show the ability of the method for correct ranking [26]. However, it is important to keep in mind that correlation coefficients do not measure agreement but rather show if a method ranks correctly when compared with a reference method (in our case, the weighed amounts). Since Bland-Altman plot method only defines the intervals of agreements [27], but does not say whether those limits are acceptable or not, we believe it is helpful to use both methods in validation studies.

## Conclusion

In conclusion, these results suggest that the photographic method developed for the Promeal project provides useful estimates of dietary intake in terms of amount of food and special food items as well as energy intake during school meals. The method proved valid for estimation of total intake as well as for different food groups and shapes of food. The information from the photograph method can give important information on which foods children put on their plates in the school canteen and what is actually being eaten. Furthermore the method is practical since it does not interfere much with the daily routine of the personnel and children, which is important when performing research involving the school setting.

## Supporting Information

S1 Fig **Example of page in reference booklet from Iceland**; 90° (top) and 45° (bottom). Half reference portion left, full reference portion right (tif).
(TIF)

S1 File Dataset for data from Iceland. Raw data (xlsx).
(XLSX)

S2 File Dataset for data from Sweden. Raw data (xlsx).
(XLSX)
