# Supplementary figures and images for: Development and Validation of a Photographic Method to Use for Dietary Assessment in School Settings
Source: PLoS One. 2016 Oct 6;11(10):e0163970. doi: 10.1371/journal.pone.0163970 (PMC5053534; doi:10.1371/journal.pone.0163970)

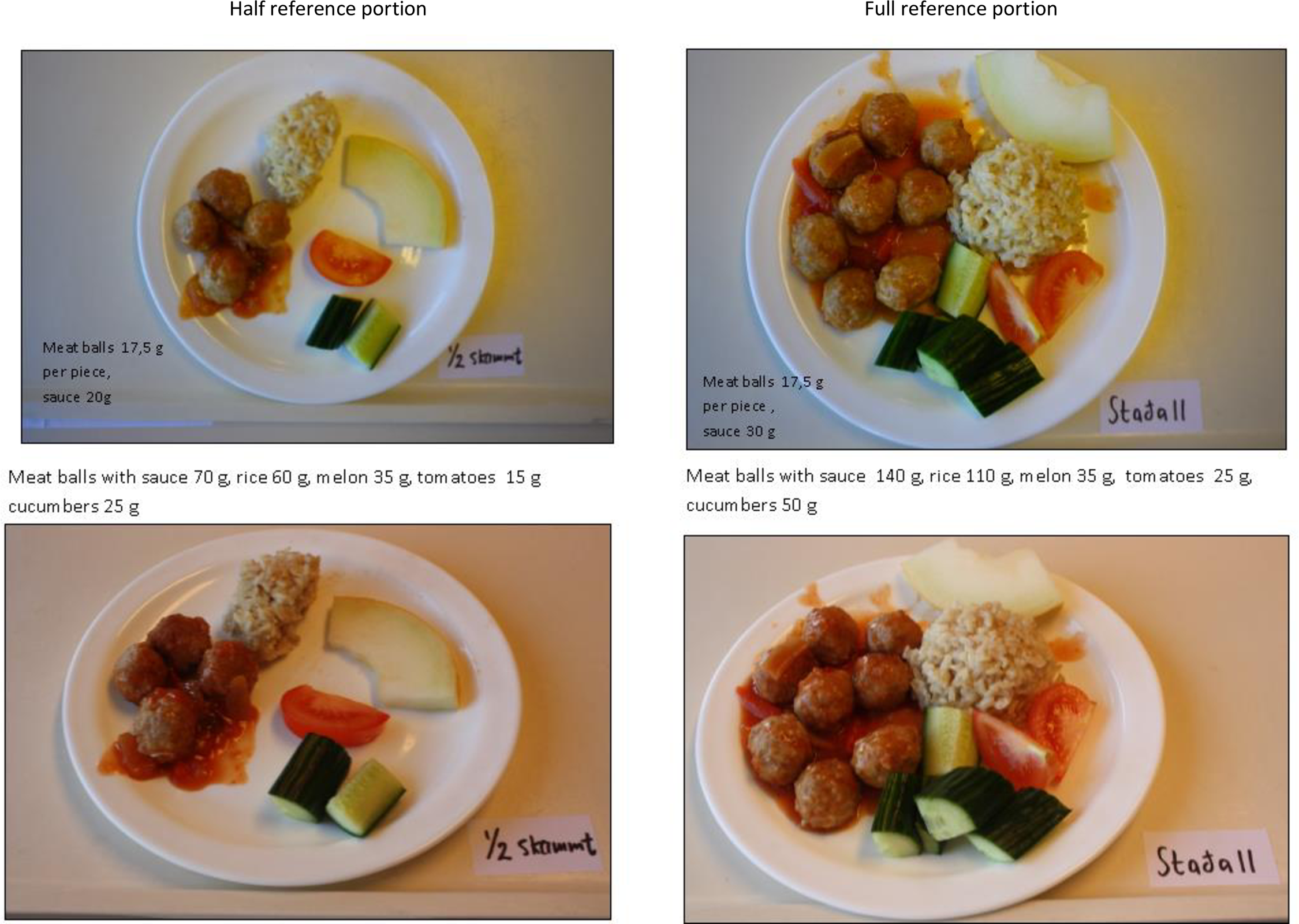

Supplement: S1 Fig — Example of page in reference booklet from Iceland; 90° (top) and 45° (bottom). Half reference portion left, full reference portion right (tif). (TIF) [file pone.0163970.s001.tif]
